# A rare case of life-threatening mixed germ cell tumor infiltrating the heart: A case report

**DOI:** 10.1016/j.ijscr.2023.108385

**Published:** 2023-06-09

**Authors:** Muhammad Hilmy Ayundra, Navy Laksmono, Tri Wahyuni Murni

**Affiliations:** a Department of Surgery, Faculty of Medicine, Padjadjaran University, Dr. Hasan Sadikin General Hospital, Bandung, Indonesia; b Division of Cardiac and Thoracic Surgery, Department of Surgery, Faculty of Medicine, Padjadjaran University, Dr. Hasan Sadikin General Hospital, Bandung, Indonesia

**Keywords:** Mixed germ cell tumor, Mediastinum mass, Heart infiltration, Cardiothoracic surgery

## Abstract

**Introduction and importance:**

Extragonadal germ cell tumors at the mediastinum are rare and comprise of 3–4 % of all germ cell tumors. Mixed GCTs can remain asymptomatic for long periods and often present with complications. We present a case of a young male patient with a mediastinal tumor infiltrating the heart and obstructing the right ventricular outflow tract, causing cardiogenic shock.

**Case presentation:**

A 16-year-old male came with chief complain of shortness of breath and underwent an echocardiogram which revealed a mass in the right atrium and right ventricle. On CT scan, a solid mass in the mediastinum, expanding and infiltrating the right atrium was found. Our patient underwent surgical treatment. Histopathology results were consistent with mixed germ cell tumor comprised of seminoma, yolk sac, and mature teratoma at the right atrial and mediastinum.

**Clinical discussion:**

The pathogenesis of extragonadal GCTs has been linked to abnormal and/or incomplete migration of the primordial germ cells from the endoderm yolk sac to gonads. Mediastinum GCT can become clinically problematic through its growth patterns, especially its expansive profile, which can cause compression on surrounding mediastinal structures, including major vessels, which in turn diminish blood flow. Overall survival improvement is strongly linked with surgical resection of the tumor, which achieve removal of tumor tissue resistant to chemotherapy and provides sample for histological examination, which helps assessment of pathological response to chemotherapy and planning of further management.

**Conclusion:**

The mediastinum is a site of different neoplasia, including germ cell tumors. Despite its low incidence, the diagnosis of a mediastinal mixed germ cell tumor should be considered in young patients with a mediastinal mass. This tumor is aggressive and often infiltrates surrounding structures and metastasis. Physicians must be aware of the difficulties and complications associated with the diagnosis.

## Highlights

• Mixed germ cell tumors of the anterior mediastinum are a sporadic and aggressive disease that often invades and infiltrates surrounding structures and metastasis. These tumors can present in a life-threatening condition, such as cardiogenic shock.
• These tumors can get big enough to compress on mediastinal structures leading to a medical emergency.
• Primary GCT of the mediastinum often has a poor prognosis.
• Surgery contributed to long-term survival as part of a multimodal aggressive treatment approach.
• A better understanding of this disease's clinical and imaging knowledge is the key to early diagnosis, early intervention treatment, and improved prognosis.

## 1 Introduction

Germ cell tumors (GCTs) typically originate from the gonads, more frequently from the testes than ovaries, and are often found in adolescents and young adults [1]. Extragonadal GCTs are rare; even more rare are those located in the mediastinum, which account for only 3 %–4 % of all GCTs [2]. Mixed GCTs of the mediastinum are neoplasms composed of two or more types of GCT [3]. Mixed GCTs can remain asymptomatic for long periods and often present with complications [4].

We present a rare case of a life-threatening mixed GCT of the mediastinum that infiltrated the heart causing cardiogenic shock in a 16-year-old male patient.

## 2 Case report

**Fig. 1:**
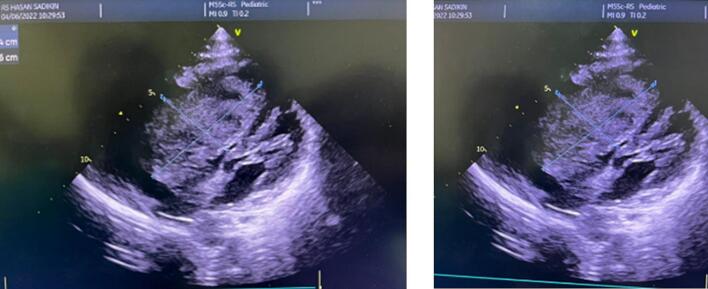
Transthoracic echocardiography reveals a mass filling the right atrium and right ventricle at PA.

A 16-year-old male patient came to the hospital complaining of shortness of breath in 1 month, worsening two weeks before admission. Complaints get worse if the patient is in a supine position or with strenuous activities. The patient also complained of chest pain, nausea, dizziness, and fatigue. The patient has no fever, no history of prolonged cough, and no coughing up blood. Because of his complaint, the patient came to the nearest hospital for four days. The patient underwent echocardiography examination which revealed a myxoma extending from the right atrium to the right ventricle. He was then transferred to our hospital for further management. A repeat echocardiography was conducted at our hospital, which confirmed these findings, showing a mass filling the right atrium and right ventricle seen at PA with minimal pericardial effusion at the anterior and posterior (Fig. 1).

The patient's vital signs findings include tachycardia (heart rate 120 beats per minute), tachypnea (respiratory rate of 34 breaths per minute), and hypoxemia (oxygen saturation of 78 % on room air). There were no abnormalities on the physical examination and no pathological murmurs in areas of valve auscultation. Laboratory examination may show non-specific changes such as increased hemoglobin, hematocrit, leukocytosis, thrombocytopenia, hyponatremia, hyperkalemia, and hypocalcemia. Liver function tests showed an increase in SGOT and SGPT. Blood gas analysis revealed increased blood pH and decreased pO2, pCO2, HCO3, and base excess.

**Fig. 2:**
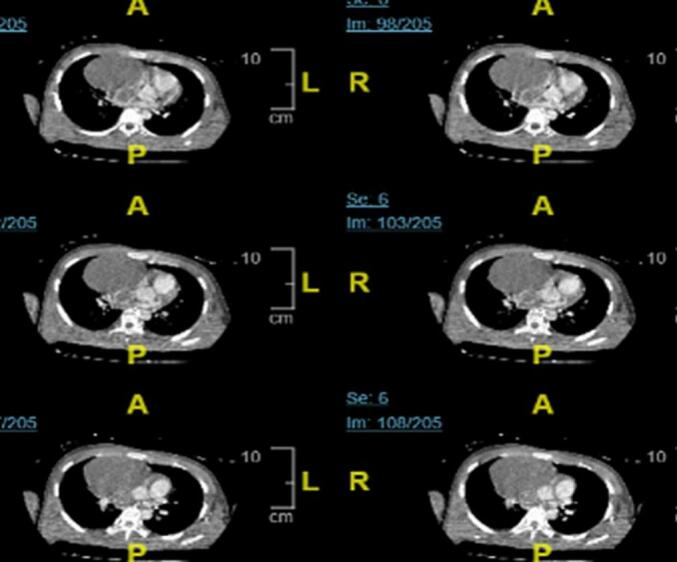
CT scan showed a solid mass in the superoanterior mediastinum, expanding and infiltrating the right atrium.

Chest computed tomography (CT) scan revealed a solid mass in the superoanterior mediastinum, which appeared to be attached to the brachiocephalic trunk, ascending aorta, right pulmonary artery, compressing and constricting the superior vena cava, and expanding and infiltrating the right atrium, suspected lymphoma (Fig. 2).

**Fig. 3:**
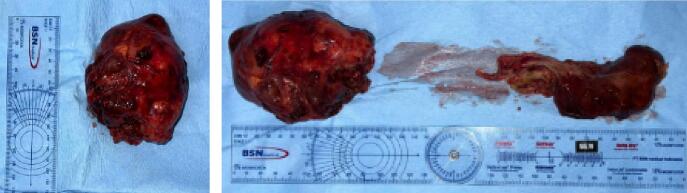
The macroscopic aspect of the resected tumor mass appears irregular and well-defined.

**Fig. 4:**
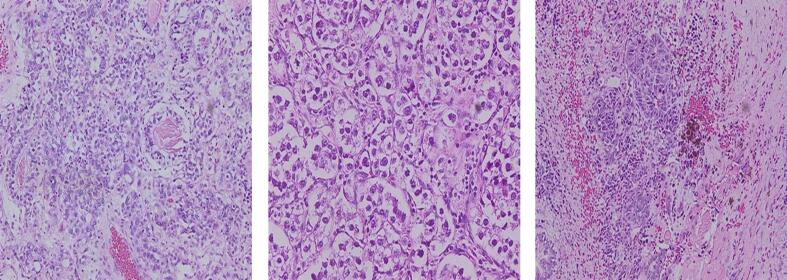
Histopathologic examination showed the tumor cells have a pleomorphic nucleus. The schiller-Duval body was seen.

Because of the unstable hemodynamics, the patient underwent the removal of the tumor. Following median sternotomy, anterior mediastinal tumor tissue appears to fill the entire anterior mediastinal space. The tumor was found to have invaded the innominate vein and caused extensive adhesions. The tumor was excised, the injured innominate vein was ligated, and the tumor was successfully evacuated. The pericardium is opened, and the heart appears normal in size and pericardial fluid was also within normal limits. We performed aortic cross clamp and antegrade cardioplegia to stop the heartbeat temporarily. A Myxoma mass was seen in the right atrium, originating at the roof of the SVC, and myxoma evacuation was performed (Fig. 3). The cardiopulmonary bypass (CPB) time was 107 min. On the histopathologic examination, polymorphic nuclei were found at the tumor cells. The schiller-Duval body was seen, which is pathognomonic for yolk sac tumors (Fig. 4). A histopathological study confirmed the diagnosis of a mixed germ cell tumor (seminoma, yolk sac, and mature teratoma) at the right atrial and mediastinum.

## 3 Discussion

Germ cell tumors (GCTs) are approximately 15 %–20 % of all anterior mediastinal masses, occurring most often in the ages of 20 to 40 years, with equal distribution on both male and female. Most of GCTs are benign, with teratoma being the most common type [5]. Extragonadal GCTs comprise aproximately 10 % of all GCTs, mostly occuring at the midline, with the anterior mediastinum as the most frequent site [6,7].

Mediastinum GCT predominantly affects males. Increased risk of mediastinum GCT is observed in individuals with Klinefelter syndrome. Hence investigation of this syndrome in young patients with mediastinum GCT is strongly recommended [8].

GCTs are classified based on its histologic characteristics, in the similar classification as the gonads: seminomatous (pure), nonseminomatous (yolk sac tumor, embryonal carcinoma, choriocarcinoma, and mixed GCTs), and teratomas [9]. Mediastinal mixed GCTs are malignant non-seminomatous GCTs consisting of more than one type of GCT [3]. Primary mediastinal GCTs account for 10–15 % of all mediastinal malignancies and 1–3 % of all GCTs [1].

The pathogenesis of extragonadal GCTs has been linked to abnormal and/or incomplete migration of the primordial germ cells from the endoderm yolk sac to gonads. This abnormal localization may later result in malignant transformation of the midline germ cells along the urogenital ridge [10].

The spreading of the tumor to the heart may occur through four different mechanisms, including direct extension, via the bloodstream, via lymphatic drainage, and intracavitary diffusion through superior vena cava or pulmonary veins [10].

Most patients of mixed GCT present with symptoms similar to those of other mediastinal GCTs, as well as elevated serum tumor markers [3]. Cardiac metastasis can cause various clinical presentations depending on the most involved sites. While most cases are asymptomatic, pericardial effusion may occur because of an unrecognized metastatic cancer [11].

Mediastinum GCT can become clinically problematic through its growth patterns, especially its expansive profile, which can cause compression on surrounding mediastinal structures [8]. This characteristic could cause clinical symptoms of pain or discomfort in the chest and back, cough, shortness of breath, palpitations, and associated symptoms related to tumor oppression on surrounding tissues [7]. More severely, mediastinum GCTs can cause oppression on major vessels, which in turn diminish blood flow, for example superior vena cava syndrome can occur on account of superior vena cava compression, causing a medical emergency. Pressure by the force of tumor compression can also impair small blood vessels, causing tissue necrosis, for example bronchial wall necrosis can result in hemoptysis indicating airway perforation [8].

Increased values of serum alpha phetoprotein (AFP), human chorionic gonadotropin (HCG), and lactate dehydrogenase (LDH) might be seen in mixed GCT patients [7]. Imaging studies such as CT scans typically show a large, heterogeneous mass with necrosis, hemorrhage, and often infiltration of adjacent structures [3].

Histopathological examination is vital for the diagnosis of mixed GCTs [8]. On macroscopic appearance, mixed germ cell tumors are resected after chemotherapy. The tumors show a heterogeneous cut surface, with solid fleshy tumors interspersed with regions of hemorrhage and necrosis. The presence of cystic spaces usually indicates the presence of a teratomatous component [3]. Histopathologically, various GCTs can occur in any combination, and their morphologies are identical to those of GCTs [3].

Chemotherapy and subsequent surgical resection of persistent disease is the current standard treatments for patients with mediastinal mixed GCTs, which is required in >95 % patients [6]. A critical factor in the prognosis of mediastinal mixed GCTs is complete surgical resection [7]. In all cases, surgery technique largely depends on the GCT type, aiming for complete excision [12].

Contacts between mediastinal GCTs and surrounding structures may be further complicated by connections in between fibrous bands or infiltrative growth patterns of neoplastic cells. In these cases, surgical interventions consist of removal of infiltrated organs together with the tumor [8]. Since anterior mediastinum is the most frequent location of the tumor, the surgical approach of anterolateral or posterolateral thoracotomy, or median sternotomy are commonly used [12].

Overall survival improvement is strongly linked with surgical resection of the tumor, which achieve removal of tumor tissue resistant to chemotherapy and provides sample for histological examination, which helps assessment of pathological response to chemotherapy and planning of further management [12]. However, most patients may not have the opportunity for surgical intervention, owing to a larger tumor, embeded vessels, and distant metastasis at diagnosis. Chemotherapy regimens of EP (etoposide plus cisplatin) and paclitaxel plus ifosfamide plus cisplatin are recommended for these patients [7]. Chemotherapy after residual mass resection is essential to control the GCT morbidity [13].

Mixed germ cell tumors have an unfavorable prognosis. The overall 5-year survival rate is 45 %. The most favorable outcomes are in patients aged <30 years with the localized disease to the mediastinum and normal hCG levels. Children have a better prognosis [3].

## 4 Conclusion

Mixed GCTs of the anterior mediastinum are a sporadic and aggressive disease that often invades and infiltrates surrounding structures and metastasis. These tumors can present in a life-threatening condition, such as cardiogenic shock, prompted by an increase in size causing compression on mediastinal structures leading to a medical emergency. Primary GCT of the mediastinum often has a poor prognosis, which can be improved through deeper knowledge of this disease's clinical manifestations and imaging findings, so as an early diagnosis and multi-disciplinary intervention might be accomplished.

## Consent

Written informed consent was obtained from patient about these case reports writing and publishing. The patient understood well and gave consent. A copy of the written consent is available for review by the Editor-in-Chief of this journal on request.

## Underlying data and material

All supporting data are available within this study.

## Ethical approval

As it is a case report, ethical approval is exempted by our institution: Dr. Hasan Sadikin Hospital Bandung and Padjadjaran University.

## Funding

None declared.

## Author contribution

Conception and design of study, acquisition of data, analysis and interpretation of data, drafting the manuscript, revising the manuscript critically for important intellectual content, approval of the version of the manuscript to be published: Muhammad Hilmy Ayundra, Navy Laksmono, Tri Wahyuni Murni.

All authors have read and agreed to the final manuscript.

## Guarantor

Muhammad Hilmy Ayundra acts as the guarantor of this study.

## Research registration number

Not applicable.

## Declaration of competing interest

All authors declare that there is no conflict of interest regarding the publication of the article.
